# Stewardship According to Context: Justifications for Coercive Antimicrobial Stewardship Policies in Agriculture and their Limitations

**DOI:** 10.1111/bioe.13292

**Published:** 2024-04-20

**Authors:** Tess Johnson

**Affiliations:** Ethox Centre, Nuffield Department of Population Health, University of Oxford, United Kingdom

**Keywords:** Antimicrobial stewardship, public health ethics, coercion, context-specificity, antibiotic use in livestock

## Abstract

Antimicrobial resistance (AMR) is an urgent, global threat to public health. The development and implementation of effective measures to address AMR is vitally important but presents important ethical questions. This is a policy area requiring further sustained attention to ensure that policies proposed in National Action Plans (NAPs) on AMR are ethically acceptable, and preferable to alternatives that might be less coercive or more effective, for instance. By ethically analysing case studies of coercive actions across countries, we can better inform policy in a context-specific manner. In this paper, I consider an example of coercive antimicrobial stewardship policy in Canada, namely restrictions on livestock farmers’ access to certain antibiotics for animal use without a vet’s prescription. I introduce and analyse ethical arguments that might plausibly justify coercive action in this case, including the harm principle and a duty of collective easy rescue. In addition, I consider the factors that might generally limit the application of those ethical concepts, such as challenges in establishing causation or evidencing the scale of the harm to be averted. I also consider specifics of the Canadian context in contrast to the UK and Botswana as example settings, to demonstrate how context-specific factors might mean a coercive policy that is ethically justified in one country is not so in another.

## Introduction

Antimicrobial resistance (AMR) is an urgent, global threat to public health. It is associated with 4.95 million deaths per year, and of those, it directly causes around 1.27 million deaths per year.^1^ In 2019, AMR killed more people than malaria or HIV/AIDs.^2^ AMR arises when micro-organisms develop resistance to the drugs (antibiotics and other antimicrobials) designed to kill them. Often, these resistant micro-organisms go on to cause disease in animals and humans, and changes to ecosystems. AMR is a natural phenomenon, the result of evolutionary adaptation by micro-organisms, and so it is, to some extent, inevitable—but it is also exacerbated by the misuse of antimicrobials. Antimicrobial stewardship—that is, optimising use of antimicrobials, and particularly reducing the use of drugs that offer a last line of defence against dangerous pathogens—is a key method of protecting the ‘antimicrobial commons’, the globally shared resource that is continued antimicrobial effectiveness.^3^ Stewardship may require different kinds of actions by states, institutions and individuals in countries where overuse (i.e., excess) of antibiotics is more of a problem than underuse (i.e., access). In general, many high-income countries (HICs) are at greater risk of excessive use, and often it is low- and middle- income countries (LMICs) that are at greater risk of inadequate access to antibiotics when needed.

Antimicrobials cross human, animal and environmental reservoirs in a number of different ways. Often, this is through human medical use of antimicrobial drugs and subsequent antimicrobials in human waste, or antimicrobial pollution from pharmaceutical manufacturing of those drugs.

Another way that AMR spreads within and across reservoirs is through animals. Animals are often fed antibiotics to promote their growth, to prevent disease (prophylaxis), or to treat disease in individuals or across a whole herd. The meat from these animals can be contaminated with antibiotics or can contain resistant pathogens that might spread to humans if the meat is insufficiently cooked.^4^ Animal waste products used as fertiliser can further spread antibiotics or resistant pathogens in the agricultural environment.^5^ With multiple different pathways toward resistance and adverse outcomes for human and animal health and environmental resilience, the use of antibiotics in animals is an area where stewardship is particularly critical, and reduction efforts might be particularly effective at managing resistance.

Coercive policies might reduce overuse of antimicrobials. Coercion occurs when a coercer (in this case, usually a state department, agency or regulatory body) uses threat or force on a coercee (in this case, either an individual or a group within the remit of state policy) in order to ensure compliance with an action. ^6^ There are three common views on the moral status of coercion. Whilst some scholars hold that coercion is intrinsically wrongful,^7^ often, policymakers and the public feel that coercion might be *pro tanto* wrong, but that coercive policies can be ethically justified under some circumstances, such as during public health crises. In these cases, ethical justifications such as preventing harm to the coercee or to others might justify coercion.^8^ The final possible view is that coercion is morally neutral. Under either of the latter two views, coercive policies for antimicrobial stewardship may be morally acceptable, but under the *pro tanto* wrong view, the policy will require some form of ethical justification that outweigh the wrong of coercion in order to be ethically acceptable overall. Under the final view, no special moral justification is required. In a desire to address commonly expressed public and political concerns with coercion, I adhere for my purposes here to the dominant view, that coercion is *pro tanto* wrong, but can sometimes be justified,^9^ I hold that, alongside an ethical justification being analysed, any ‘limiting factors’ should also be considered—that is, limits to the circumstances under which the justifications apply. In policy, these justifications and their limiting factors are often opaque or not investigated,^10^ which poses two problems. First, it limits the potential motivational force behind policy, which is better delivered through policy with clear ethical justification that is appropriate to the jurisdiction. Second, it prevents the public from reflecting on the alignment between policy interventions and their ethical reasoning, which undermines accountability and transparency.

In this paper, I particularly rely on the case study of restrictions on antibiotic use in Canadian livestock. I explore how this policy interacts with contextual factors, which might include geography, country GDP, and history, and compare the case study to the contexts of the UK and Botswana. I conclude that the Canadian policy is ethically acceptable in the specific context of livestock farming in Canada and perhaps in the case of the UK, but this may not be the case in countries with different circumstances, particularly in relation to health and wealth, including Botswana. The same process of ethical evaluation might be undertaken for different stewardship policies in different contexts to ensure ethically acceptable efforts to manage AMR.

## Case Study: Restricting Antibiotic Use in Livestock Farming in Canada

Countries like the US and Canada have historically had significant antibiotic use in livestock farming, both to treat and prevent disease, and to promote growth. Animals reared for food consumed around 80% of all antimicrobials taken in the US in 2010,^11^ and whilst antibiotic sales for use in food animals hit a low in 2017, they have increased since then.^12^ As a result, Canada has renewed its focus on antimicrobial stewardship in livestock farming, and might provide an appropriate case study for ethical analysis that might inform the approaches of other states considering coercive action, particularly those in the process of updating existing guidance.

Canada is one country that set national goals for managing AMR early on, but fell far short of them according to 2014 assessments.^13^ For instance, in that year ‘approximately 82% of antimicrobials important to human medicine were distributed and/or sold for use in food-producing animals.’^14^ In response, some sectors mobilised to develop more specific recommendations based on the new Canadian NAP,^15^ including farmers’, veterinarians’ and animal welfare professionals’ groups. Some of these recommendations were integrated into the Food and Drug Regulations, and the activities of Canada Health and provincial authorities, rendering some actions mandatory.^16^ Among the interventions outlined in these documents are a few coercive policies with the potential to impact AMR in livestock.^17^

1. List A medically important antimicrobials are restricted. Veterinarians and pharmacies must obtain a Drug Establishment License in order to import, manufacture, formulate, and dispense such drugs to animal owners. They must also comply with Good Manufacturing Practices, and report their sales annually to Health Canada.
2. In addition, all animal owners must have a prescription in order to buy mixed feed that contains List A antimicrobials, or to obtain the antimicrobials from veterinarians or pharmacists.
3. As a prescription for List A antimicrobials is needed, and as pharmaceutical companies have voluntarily agreed to reduce growth promotant claims in marketing antimicrobials in Canada, animal owners cannot use these antimicrobials for growth promotion.

The set of policies is complemented by increased standards for animal health, cleanliness and safety in the poultry farming sub-sector, as recommended by various On-Farm Food Safety Programmes now encoded in policy.^18^ The standards are enforced via provincial boards, and affect chicken, turkey, and egg producers.

To what extent are coercive policies like these justified, and to what extent are they ethically acceptable in countries other than Canada, like the UK or Botswana? In the following sections, I consider justifications that may lie behind one of these Canadian policies—number 2 above—namely, the requirement that farmers have a prescription to buy feed mixed with List A antimicrobials or to obtain antimicrobials from vets and pharmacists. I then go on to briefly discuss how further contextual factors might limit our generalising from this Canadian case to other settings. I argue that whilst there may be a common bank of ethical justifications and limiting factors across policy jurisdictions, the specifics that each justification relies upon might vary such that different justifications are needed in different contexts. For instance, there may be more concern about increasing the price of meat in countries with lower food security, which might act as a counterweight or limit to an argument proposing to prevent harm to the population through less antimicrobial use in animals, which would then need to be raised in cleaner, safer settings, increasing the price of food for the population.^19^ Alternatively, there may be more feasible alternatives for ensuring animal welfare through minimising intensive farming and raising more free-range animals in geographically larger countries with more grazing land available.

## Ethical Justifications for Coercive Antimicrobial Stewardship Policy and Their Limitations

What possible ethical justifications might there be for the introduction of the Canadian coercive AMR policies, and specifically 2 above? There are a number of ethical justifications that might provide good reason for intervention, to support the case for the Canadian policy coercively limiting the use of List A antibiotics in livestock.^20^ I will briefly discuss some plausible contenders here, which I have chosen based on the wording and characteristics of the policy that seem, to me, to indicate these might be the justifications the policymakers have in mind. Each of the potential justifications I assess also has its limitations which need to be assessed for thorough policymaking. There are necessary requirements for the justifications to apply, both to the policy in question, and to the setting in question (Canada, for now, with brief analyses of the UK and Botswana settings to come below).

I begin by considering the ‘harm principle’.

### The Harm Principle

First, it seems that the Canadian policy might employ a justification for coercion based on the prevention of harm to others. The ‘harm principle’ was developed by J.S. Mill, holding that third parties ought only intervene in an individual’s actions when they threaten harm to others.^21^ Since then, the principle has been adapted to defend the use of coercion in criminal law and public health settings.^22^ A formalisation of the harm principle might look like this:

P1: A resident of a certain state, A, harms another resident of that state, B, if A’s action renders B’s level of wellbeing lower than it would have been, had A not taken that action.
P2: It falls within the responsibility of the state to prevent harm to its residents.
P3: Intervention by the state to prevent A’s action would prevent harm to B.
C: It falls within the responsibility of the state to intervene to prevent A’s action only where A harms B.

There are a few characteristics of the Canadian policy and its preamble in explainer documents disseminated to vets^23^ that indicate that this harm principle justification might have been used. First, in the preamble, the harms that would arise from inaction on antimicrobial resistance are discussed: ‘we will enter the post-antibiotic era, in which infectious diseases have the potential to be as devastating as they once were during the pre-antibiotics era.’^24^ There are also indications that this potential harm may be attributable to antimicrobial use in livestock farming, with the explainer stating: ‘increased veterinary oversight of antimicrobial use is needed to help decrease improper and unnecessary use of these important drugs, while still protecting animal health and food safety.’^25^

As implied in documents associated with this policy, the harm principle might justify coercive state intervention here insofar as there is the potential for harm to be caused to residents of Canada through the production of meat that might contain antibiotics or resistant pathogens, which might then cause drug-resistant diseases to emerge or spread. These harms might plausibly justify restricting farmers’ access to List A antibiotics.

For the harm principle to justify coercive action, it must fully apply to the case at hand. There might be limits to when it does apply. For instance, it must be empirically true that A’s action renders B’s level of wellbeing lower—i.e., that Canadian farmers using antibiotics without a prescription do indeed cause or risk causing harm to other people. There is evidence both for and against this causal link between antibiotic use in livestock and human health outcomes,^26^ but there is a growing bank of evidence that this causal link does exist,^27^ as reinforced by WHO guidance on antibiotic use in livestock.^28^ We must also establish that the state is the right third party to intervene in farmers’ actions. This relies on the state being legitimate and responsible for preventing harm to others. This is not only an empirical matter, but also rests on political theory. Citizens’ or residents’ expectations of state action often include guaranteeing and protecting certain basic human rights, alongside requirements such as upholding the rule of law, democratic election, and the provision of other services. The Canadian state appears to fulfil all or a majority of these requirements. It is rational for residents to hold such a state to be legitimate, and many of these aspects of protection will involve preventing the imposition of harms by others. Thus, it is plausible that preventing harms from AMR in livestock falls within the responsibility of the state.

The final part of the harm principle justification that we might question is whether coercive action will be effective at reducing harms posed by farmers’ antibiotic use. If limiting farmers’ access to List A antibiotics were ineffective at reducing antibiotic use because all use by farmers was in fact for purposes of treating sick animals, then the policy might not have the effect of preventing harm to Canadians. What’s more, there is another group whose wellbeing must be considered to ensure that coercive intervention is in fact justified, and this is the farmers themselves. If limiting antibiotic use has the effect of putting farmers at additional risk of exposure to animals with infectious diseases, then this may be a harm to them that must be considered in comparison to the harm to other Canadian residents that is prevented. In addition, the harm to profits must be considered (though we might anticipate this to be unlikely to negatively affect wellbeing more than the harms to Canadian residents from negative health outcomes associated with antimicrobial resistance). The levels of harm and the effectiveness of limiting antibiotic use to vet prescription-only use needs to be established for the justification from the harm principle to hold.

### The Duty of Collective Easy Rescue

A second concept justifying the coercive Canadian policy might refer to farmers’ duty of collective easy rescue. This duty exists where an individual or group need only make a small sacrifice in order to greatly benefit for others. In the public health ethics literature, it has been held that where this duty exists, state action may be justified to enforce fulfilment of the duty.^29^ A formalisation of the duty of collective easy rescue might look like this:

P1: There are some cases where an individual or group, A, can act to prevent harm to, or benefit, a group of people, B.
P2: In some of these cases, the cost of acting is reasonably bearable by A.
P3: In some of these cases, the cost of A’s acting is also small in comparison to the harm prevented or benefit bestowed upon B.
P4: Where there is net benefit produced by A’s acting, without unreasonable cost to them, they have a moral obligation to act.
C: From P1, P2, P3, and P4, in some cases, A has a moral obligation to act.

There are a few characteristics of the Canadian policy that point to the duty of collective easy rescue as justification. First, the preamble mentioned earlier discusses the harms to others from antimicrobial resistance, from which they might be rescued (thereby providing them benefit). Earlier in the explainer for vets,^30^ the scale of the problem of AMR as projected to 2050 is compared to other threats to human health, such as cancer, diabetes, and traffic accidents. Mortality from AMR is projected to be higher than each of these causes if the problem is not sufficiently addressed. This seems like a case of serious, imminent harm to a large group, commensurate with the requirements for the duty of easy rescue to apply. Second, the policy clearly places burdens on various stakeholders, but not ones that seem disproportionate. Veterinarians and pharmacists must seek additional licensing and will have more clientele; livestock farmers must request prescriptions for using antimicrobials; livestock farmers lose the growth promotion benefits and resulting additional profits from meat sales associated with antibiotic use. None of these burdens seem significant in comparison to projected mortality from AMR.

The duty of collective easy rescue may face some limitations in its applicability as a justification. First, it requires a link between A’s acting and benefits to B. So, it would need to be established that Canadian farmers seeking prescriptions for all uses of List A antibiotics in their livestock benefit residents of Canada (by reducing the likelihood of disease associated with resistance). This seems plausible on the basis of the same evidence informing the harm principle justification where a link was established between famers’ use of antibiotics and AMR in the human population.

Another requirement for the justification to hold is that there is an appropriate burden – benefit ratio—that is, that farmers do indeed only make a small sacrifice (in absolute and comparative terms) to greatly benefit Canadian residents. This means that both the level of benefit and the level of cost to farmers from acting must be established, as well as the appropriate ratio between them to ensure the cost to farmers is not ‘significant’ by comparison. This would mean establishing the change in the level of resistance from farmers seeking prescriptions for List A antibiotic use, and establishing the costs they experience from seeking prescriptions, which might include profit loss, their animals suffering or their having to spend more on better farming conditions, and/or their own greater risk of exposure to infections. These burdens should be small not only in comparison to the benefit to Canadian residents, but when considered alone. (Otherwise, extremely large benefits to Canadian residents could justify too much harm to farmers.) What level of profit can farmers be reasonably expected to sacrifice? What level of animal suffering or personal risk can they be expected to take on? These thresholds pose another limitation to the applicability of the duty of collective easy rescue as a justification for the Canadian policy.

What are the normative implications of my analysis of the ethical justifications explored here? There is little point in describing and analysing the fulfilment of argument premises without assessing what the arguments mean in context. In this case, it seems that both the harm principle and the duty of collective easy rescue hold, and therefore provide ethical justification for the Canadian policy. Canadian farmers indeed *should* be restricted from accessing List A antibiotics for animal use without a prescription. In the next section, I explore whether this means the policy is justified in the UK and Botswana contexts.

## Context-Specific Limiting Factors

At the May 2015 World Health Assembly, the World Health Organization (WHO) introduced a global action plan on AMR.^31^ Around the same time, it highly encouraged the development of NAPs. Many countries are currently in the process of renewing their NAPs. For instance, whilst the goals expressed in the UK’s NAP were enforced partly through EU policy up until recently, post-Brexit UK policies have diverged. As a result, whilst group-level antibiotic use for prophylaxis was banned in the EU from January 2022, as was the import of meat and dairy that has been produced using antibiotics to stimulate growth, in the UK, farmers can still give antibiotics to farm animals when they are not sick.^32^ The UK is in the process of producing its next NAP for 2025-2029, and may look to how other countries like Canada have developed their policy. Antimicrobial consumption in livestock is less of a problem in the UK than in North America, and it may seem counterintuitive for countries with lower levels of AMR to take lessons from those with more. However, there is still room for improvement in the UK’s and other countries’ unnecessary uses of antibiotics, and coercive policies offer a potential solution. Furthermore, post-Brexit, the UK has been increasing food trade with some countries where antimicrobial use in farming is more common, including the US, China, and Brazil.^33^

Another country currently on the road to fully implementing its NAP is Botswana. This middle-income country’s NAP is developed, but in the process of being implemented, and has yet to receive political support.^34^ Botswana was in the top quartile of countries for age-standardised, per capita AMR-associated mortality in 2019.^35^ It has also successfully taken some action against AMR already in the agricultural sector: Botswana is a major exporter of beef to European markets, and has already implemented beef export industry-specific monitoring systems for animal antibiotic treatment and antibiotic residue in meat.^36^ Alongside this monitoring, there has been investment in education for farmers. However, these measures do not necessarily reach to other areas of livestock farming. For instance, 27% of poultry in Botswana hosts multidrug-resistant species of *Campylobacter*.^37^ The use of fluoroquinolones in chickens has been associated with disease linked to resistant Campylobacter. ^38^ Restrictions on the use of fluoroquinolone—which is a human-critical antibiotic—in Botswana may help improve food safety and reduce mortality associated with antimicrobial resistance. Should Botswana, as well as the UK, use the Canadian policy as an example for reform?

Good stewardship and the choice of appropriate policy goals is highly context-dependent. In excess-prone countries where the prevalence of infectious diseases requiring antibiotic treatment is low and there is adequate access to health care, there must be more focus on reducing antibiotic use. This situation might be considered to apply in both Canada and the UK. Where there is a lack of access to antimicrobials and high prevalence of infectious diseases paired with under-resourced health care systems, there ought to be more focus on increasing appropriate access and ensuring equitable distribution. This might be the case for Botswana, at least to a greater extent than Canada and the UK. This will mean that ethical justifications for coercive antimicrobial stewardship policies like the Canadian livestock prescription policy do not necessarily apply across different national contexts.

Krockow and Tarrant explore contextual factors affecting the ethical dilemmas associated with AMR across countries. ^39^ They discuss how aspects of national culture such as risk avoidance, hierarchy, and attitudes toward uncertainty can affect doctors’ prescribing habits. In addition, they analyse how a country’s economic situation, health care system structure and geographic location can affect the extent to which medically complex situations arise. Whilst their focus is hospital settings, the same might go for vets prescribing antibiotics for animals which are, in some countries, in greater need of antibiotics due to increased infection occurrence in intensive farming conditions, which might itself be a result of financial insecurity or less well-resourced welfare support in some countries. In countries where intensive farming is more widespread (whether due to a larger meat product-consuming population, less space for agriculture, or lower profit margins for farmers), farmers may need antibiotics for their livestock regularly. Whilst it is difficult to find statistics on farmers’ incomes directly, World Bank data show that in the UK, agriculture, forestry and farming produce an added value of roughly USD 55,000 per worker annually, with that number up at USD 113,000 per worker in Canada.^40^ In Botswana, the value added per worker is USD 1,800.^41^ Lower-value farming in Botswana makes it much harder for farmers to increase their income.

Finally, the situation of the general population must be considered in a country-specific manner, accounting for the threat antimicrobial resistance poses in comparison to other health and non-health concerns and values. For instance, in Botswana, HIV is still endemic, with a prevalence of over 20% in the adult population, posing a large additional health burden.^42^ Whilst the country has successfully built on a foundation of diamond mining and prudent fiscal policies to become one of the fastest growing economies in the world recently, it faces high unemployment (at 26%)^43^ and receives a food security score of 51/100 according to USAID research.^44^ In countries where food security is of greater concern, there may be less room for increased food prices that might reflect reduced agricultural antibiotic use through higher animal mortality from infectious disease, loss of growth promotion through antibiotics, and less intensive farming practices to reduce the need for antibiotics in livestock.^45^ Whilst AMR resulting from antibiotic use in livestock can also contribute to food insecurity or unsafety,^46^ failures to consider the factors on both sides of the equation exacerbate existing inequalities between food-secure and food-insecure nations and can lead to the blanket promotion of policies that contribute to global injustice when implemented in contexts where they aren’t appropriate.^47^

Botswana is a middle-income country, and the ethical justifications for coercive antimicrobial stewardship policy may be more applicable in this country than others in the region. However, there is more justification for coercive stewardship in the UK and Canada, where food insecurity and other health burdens are less of a concern, and where there is more structural support and financial capacity for change in the agricultural sector.

Due to these context-specific complexities that may arise, it is essential that policymakers of a jurisdiction assess *all* the limiting factors for proposed ethical justifications of coercive antimicrobial stewardship policy thoroughly. There is fearsome potential for coercive policies advocated in HICs to become a tool in a colonial development narrative that is imposed on LMICs and that exacerbates inequalities in the burdens associated with antimicrobial stewardship efforts across countries.^48^ For this to be avoided, ethical subsidiarity is necessary: policies should be designed and implemented by the communities they will affect, without the imposition of generalisations or justifications for policies that apply in high-income settings without consideration of the limiting factors and how these may vary in a context-specific way.^49^

The following quote summarises my point in this section perfectly:

“The importance of country-owned and developed national action plans to combat AMR using appropriately tailored interventions cannot be overstated. […] In our opinion, many other National Action Plan (NAP) pillars under-exploit opportunities for impact because they prioritize top-down interventions aimed at combatting resistance in high-income settings that may not always be suitable for or effective in other parts of the world […].” ^50^

## Conclusion

Protecting human and animal health and environmental resilience depends on our optimising antimicrobial use in the coming decades. An important part of this is reducing use in sectors where antibiotics are consumed inappropriately, exacerbating AMR.

Agriculture is one area where coercive policies have appeared to have promising results. In Canada, policy limiting use of List A antimicrobials by farmers without veterinarian prescriptions provides a good example of ethically justified coercive policy for that context. The policy can be defended by reference both to the harm principle and the duty of collective easy rescue.

However, these justifications are limited in a general way according to, for instance, the availability of evidence of causal links between farming practices and worse human health outcomes from AMR. More importantly, they are highly limited in their applicability to other national contexts: whilst the policy might be used as an example for the UK context, other antimicrobial stewardship and access measures may be more appropriate in the Botswana setting. Generalisations across countries may render invisible morally relevant country-specific concerns that may render some coercive stewardship interventions less (or un-)justifiable. It is essential that policies are co-designed at national, state or local levels by and for the communities that will be affected by them. Only by thoroughly considering ethical justifications for coercive policy and their limitations in a context-specific manner can we acceptably and appropriately tackle the global problem of antimicrobial resistance.

## Footnotes

1 Interagency Coordination Group on AMR. (2019). *No time to wait: securing the future from drug-resistant infections*. Retrieved from https://www.who.int/docs/default-source/documents/no-time-to-wait-securing-the-future-from-drug-resistant-infections-en.pdf (pdf).

2 Murray, C.J.L., Ikuta, K.S., Sharara, F., Swetschinski, L., Aguilar, G.R., Gray, A., … Naghavi, M. (2022). Global burden of bacterial antimicrobial resistance in 2019: A systematic analysis. *The Lancet*. 399(10325), 629-655.

3 Giubilini, A. (2019). Antibiotic resistance as a tragedy of the commons: An ethical argument for a tax on antibiotic use in humans. *Bioethics*, 33(7), 776-784.

4 Rega, M., Andriani, L., Poeta, A., Bonardi, S., Conter, M., & Bacci, C. (2023). The pork food chain as a route of transmission of antimicrobial resistant Escherichia coli: A farm-to-fork perspective. *Antibiotics (Basel)*. 12(2), 376.

5 Marutescu, L.G., Jaga, M., Postolache, C., Barbuceanu, F., Milita, N.M., Romascu, L.M., … Popa, M. (2022). Insights into the impact of manure on the environmental antibiotic residues and resistance pool. *Frontiers in Microbiology*. 13, 965132.

6 Anderson, S. (2021). Coercion. In E.N. Zalta, *Stanford Encyclopedia of Philosophy*. Retrieved from https://plato.stanford.edu/archives/sum2021/entries/coercion/.

7 Wertheimer, A. (1987). *Coercion*. Princeton: Princeton University Press.

8 For instance, in the public health context, and Savulescu (2020) consider there to be a surmountable presumption against coercive policies that would restrict patient access to antimicrobials, however, they think this can be overcome when one considers a policy limited to coercing patients who are not in great need of the antibiotics, because this falls under a ‘duty of easy rescue’. Giubilini A., & Savulescu, J. (2020). Moral responsibility and the justification of policies to preserve antimicrobial effectiveness. In E. Jamrozik & M. Selgelid, *Ethics and drug resistance: Collective responsibility for global public health*. Cham: Springer International Publishing, pp. 141-154.

9 For a public health policy and ethics example of this conception, see the Nuffield Council on Bioethics’ public health intervention ladder, which takes an initial stance on public health interventions primarily according to their coerciveness. Nuffield Council on Bioethics (2007). *Public health: ethical issues*. Nuffield Council on Bioethics: London, UK. I do not, however, hold that this is the best view, theoretically. Per Anderson (2010), I hold with a morally neutral view of coercion. However, addressing the dominant view is important so long as it remains a concern for public and policymakers whose actions are required to address AMR. What’s more, insofar as all public health policies require ethical justification, coercive measures are one group which often share appropriate ethical justifications, such as those I present here. Anderson, S. (2010). The enforcement approach to coercion. *Journal of Ethics and Social Philosophy*, 5(1), 1-31.

10 Smith, M.J., & Upshur, R. (2020). Learning lessons from COVID-19 requires recognizing moral failures. *Journal of Bioethical Inquiry*. 17(4), 563-566.

11 Food and Drug Administration. (2009). *2009 Summary report on antimicrobials sold or distributed for use in food-producing animals*. Retrieved from https://www.fda.gov/media/79581/download (pdf).

12 Food and Drug Administration. (2020). *2020 Summary report on antimicrobials sold or distributed for use in food-producing animals*. Retrieved from https://www.fda.gov/media/154820/download?attachment (pdf).

13 Ad-Hoc Committee for Antimicrobial Stewardship in Canadian Agriculture and Veterinary Medicine. (2014). Stewardship of antimicrobial drugs in animals in Canada: How are we doing? *Canadian Veterinary Journal*. 55(3), 273-276.

14 Public Health Agency of Canada. (2017). *Tackling antimicrobial resistance and antimicrobial use: A pan-Canadian framework for action*. Retrieved from: https://www.canada.ca/en/health-canada/services/publications/drugs-health-products/tackling-antimicrobial-resistance-use-pan-canadian-framework-action.html. Quote at p. 19.

15 Ibid.

16 Government of Canada. (2017). *Regulations amending the food and drug regulations (veterinary drugs — antimicrobial resistance)*. Retrieved from https://gazette.gc.ca/rp-pr/p2/2017/2017-05-17/html/sor-dors76-eng.html

17 Farmed Animal Antimicrobial Stewardship (2019). *Veterinarian reference manual*. Retrieved from https://www.amstewardship.ca/faast-manuals-available-for-download/.

18 Farmed Animal Antimicrobial Stewardship (2019). Antimicrobial stewardship in food animals in Canada: Progress on recommendations and stakeholder activities. Retrieved from https://www.amstewardship.ca/antimicrobial-stewardship-in-food-animals-in-canada-progress-on-recommendations-and-stakeholder-activities/.

19 This is not to say that such a measure might not be, on the whole, justified, but merely that when considering the prevention of harm to others, there are many context-specific harms other than exposure to a resistant pathogen that must be considered.

20 Note that in considering various ethical justifications here, I take an implicitly principlist approach. I explore moral principles that might appeal to a broad audience, and that avoid alienating large swathes of decision-makers, the way that a more traditional theory-driven approach like a utilitarian or Kantian ethical analysis might do.

21 Mill, J.S. (1859). *On Liberty* (second edition). London: John W Parker and Sons.

22 Feinberg, J. (1984). Harm to others. In *The moral limits of the criminal law*. Oxford: Oxford University Press, volume 1; Griffiths, P., & West, C. (2015). A balanced intervention ladder: promoting autonomy through public health action. *Public Health*. 128(8), 1092-1098.

23 Farmed Animal Antimicrobial Stewardship, op. cit. note 23.

24 Ibid, p. 2.

25 Ibid, p. 40.

26 Hoelzer, K., Wong, N., Thomas, J., Talkington, K., Jungman, E., & Coukell, A. (2017). Antimicrobial drug use in food-producing animals and associated human health risks: What, and how strong, is the evidence? *BMC Veterinary Research*. 13, 211.

27 Tang, K.L., Caffrey, N., Nobrega, D., Cork, S., Ronksley, P., Barkema, H., … Ghali, W. (2017). Restricting the use of antibiotics in food-producing animals and its associations with antibiotic resistance in food-producing animals and human beings: a systematic review and meta-analysis. *The Lancet Planetary Health*. 1(8), e316-e327. See also: Pirolo, M., Visaggio, D., Gioffrè, A., Artuso, I., Gherardi, M., Pavia, G., … Visca, P. (2019). Unidirectional animal-to-human transmission of methicillin-resistant Staphylococcus aureus ST398 in pig farming; evidence from a surveillance study in southern Italy. *Antimicrobial Resistance and Infection Control*. 8, 187.

28 World Health Organization. (2017). *Stop using antibiotics in healthy animals to prevent the spread of antibiotic resistance*. Retrieved from https://www.who.int/news/item/07-11-2017-stop-using-antibiotics-in-healthy-animals-to-prevent-the-spread-of-antibiotic-resistance

29 Giubilini, A., Douglas, T., Maslen, H., & Savulescu, J. (2018). Quarantine, isolation and the duty of easy rescue in public health. *Developing World Bioethics*. 18: 182–189.

30 Farmed Animal Antimicrobial Stewardship, op. cit. note 23.

31 World Health Organization. (2016). *Global action plan*. Retrieved from https://www.who.int/publications/i/item/9789241509763.

32 Levitt, T. (2022, January 28). UK risks falling behind on reducing farm antibiotics after EU ban. *The Guardian*. Retrieved from https://www.theguardian.com/environment/2022/jan/28/uk-risks-falling-behind-on-reducing-farm-antibiotics-after-eu-ban.

33 Gerval, A., and Jelliffe, J. (2023 December 5). Since Brexit, United Kingdom’s Agricultural Trade With European Union Remains Strong; Opportunities for U.S. Exports Emerge. *Amber Waves (The Economic Research Service, US Department of Agriculture)*. Retrieved from https://www.ers.usda.gov/amber-waves/2023/december/since-brexit-united-kingdom-s-agricultural-trade-with-european-union-remains-strong-opportunities-for-u-s-exports-emerge/.

34 World Health Organization. (2021). *Botswana: 2021 TrACSS Country Report on the Implementation of National Action Plan on Antimicrobial Resistance (AMR)*. Retrieved from https://cdn.who.int/media/docs/default-source/antimicrobial-resistance/amr-spc-npm/tracss/tracss-2021-botswana.pdf?sfvrsn=ec87ff5d_3&download=true (pdf).

35 Institute for Health Metrics and Health Evaluation. (2023). *The burden of antimicrobial resistance (AMR) in Botswana*. Retrieved from https://www.healthdata.org/sites/default/files/files/Projects/GRAM/Botswana_0.pdf (pdf).

36 Kgakatsi, L. (2023, August 9). Botswana reinforces collaborative efforts to reduce antimicrobial resistance. *Center for Coordination of Agricultural Research and Development for South Africa*. Retrieved from https://www.ccardesa.org/botswana-reinforces-collaborative-efforts-reduce-antimicrobial-resistance.

37 de Vries, S.P.W., Vurayai, M., Holmes, M., Gupta, S., Bateman, M., Goldfarb, D., … Grant, A.J. (2018). Phylogenetic analyses and antimicrobial resistance profiles of Campylobacter spp. from diarrhoeal patients and chickens in Botswana. *PLoS One*. 13(3), e019448.

38 Ibid.

39 Krockow, E.M., & Tarrant, C. (2019). The international dimensions of antimicrobial resistance: Contextual factors shape distinct ethical challenges in South Africa, Sri Lanka and the United Kingdom. *Bioethics*. 33(7), 756-765.

40 The World Bank. (2023). *Agriculture, forestry, and fishing, value added per worker (constant 2015 US$)*. Retrieved from https://data.worldbank.org/indicator/NV.AGR.TOTL.ZS

41 Ibid.

42 Global Aids Monitoring (2020). *Country progress report – Botswana*. Retrieved from https://www.unaids.org/sites/default/files/country/documents/BWA_2020_countryreport.pdf (pdf).

43 The World Bank (2023). *The World Bank in Botswana*. Retrieved from https://www.worldbank.org/en/country/botswana/overview

44 US Aid. (2023). *Botswana: Sector detail – food security*. Retrieved from https://idea.usaid.gov/cd/botswana/hunger-and-food-security#tab-food-security

45 Vagsholm, I., Arzoomand, N., & Boqvist, S. (2020). Food security, safety, and sustainability—getting the trade-offs right. *Frontiers in Sustainable Food Systems*. 4, doi:10.3389/fsufs.2020.00016.

46 Founou, L.L., Founou, R.C., & Essack, S.Y. (2021). Antimicrobial resistance in the farm-to-plate continuum: more than a food safety issue. *Future Science OA*. 7(5), FSO692.

47 Reid, L. (2020). Antimicrobial resistance and social inequalities in health: Considerations of justice. In In E. Jamrozik & M. Selgelid, *Ethics and drug resistance: Collective responsibility for global public health*. Cham: Springer International Publishing, pp. 257-278.

48 Ibid.

49 Jecker, N.S., Atuire, C.A., & Bull, S.J. (2023). Towards a new model of global health justice: the case of COVID-19 vaccines. *Journal of Medical Ethics*. 49, 367-374.

50 Ikhimiukor, O.O., Odih, E.E., Donado-Godoy, P., & Okeke, I.N. (2022). A bottom-up view of antimicrobial resistance transmission in developing countries. *Nature Microbiology*. 7, 757–765, p. 760.

